# Tropomyosin micelles are the major components contributing to the white colour of boiled shellfish soups

**DOI:** 10.1038/s41598-022-17911-8

**Published:** 2022-09-09

**Authors:** Takashi Akihiro, Ryo Yasui, Shinji Yasuhira, Ken-ichi Matsumoto, Yasuhiro Tanaka, Yasuhiro Matsuo, Hidehisa Shimizu, Takashi Matsuzaki, Shingo Matsumoto, Keisuke Yoshikiyo, Hideki Ishida

**Affiliations:** 1 Faculty of Life and Environmental Sciences, Shimane University, 1060, Nishikawatsu, Matsue, Shimane 690-8504 Japan; 2 Institute for Biomedical Sciences, Iwate Medical University, 1-1-1 Idai-dori, Yahaba-cho, Shiwa-gun, Iwate, 028-3694 Japan; 3 Department of Biosignaling and Radioisotope Experiment, Interdisciplinary Center for Science Research, Organization for Research and Academic Information, Shimane University, Izumo, Shimane 693-8501 Japan; 4 Bioresource and Life Sciences, The United Graduate School of Agricultural Sciences, Tottori University, 4-101 Koyama-Min-ami, Tottori, Tottori 680-8553 Japan; 5 Shimane University, 1060, Nishikawatsu, Matsue, Shimane 690-0823 Japan

**Keywords:** Proteins, Molecular biology

## Abstract

Basket clam soup, a popular Asian dish, is prepared by boiling clams in hot water. The soup is generally cloudy, and it is considered that increased cloudiness enhances taste. However, the composition of the whitening ingredients and their association with taste enhancement remains unclear. In this study, we aimed to identify the components contributing to the white colour of the boiled soup. The white component upon precipitation with trichloroacetic acid reacted positively with ninhydrin, indicating the presence of proteins. The separation of proteins using sodium dodecyl sulphate–polyacrylamide gel electrophoresis revealed an intense band of size 33 kDa. Peptide mass fingerprinting of the identified protein using matrix-assisted laser desorption/ionisation-time-of-flight tandem mass spectrometry revealed the protein as tropomyosin. To validate the involvement of tropomyosin in the turbidity of the soup, tropomyosin was expressed and extracted from *Escherichia coli*. As expected, the purified protein suspended in water resulted in turbid appearance. To determine whether lipids have any association with the observed cloudiness of the soup, the amounts of fatty acids were measured. The proportion of estimated fatty acids was very low compared to that of proteins. Overall, we identified the major component contributing to soup cloudiness as tropomyosin forming micelles.

## Introduction

Basket clams are bivalves that live in freshwater and brackish water^1^. The clam species that are most widely consumed are Asian clams (*Corbicula fluminea* and *Corbicula leana*) and Japanese basket clam (*Corbicula japonica*). In 2018, approximately 32,000 tonnes of basket clams were harvested and consumed worldwide, including 18,000 tonnes in China, 9700 tonnes in Japan, and 4600 tonnes in Taiwan^2^. In the 1920s, the Asian clam was exported from southeast Asia to the western parts of North America as a food source^3^, followed by South America and Europe^4^. The fertility of Asian clam is extremely high, and *C. fluminea* is considered as one of the ‘100 worst invading species’ in Europe and is recognised as an important alien species that poses a threat to biodiversity and economy^5^. Although Asian clam is frequently consumed in Asia, it is not that commonly consumed in North America, South America, and Europe.

Ben Cao Gang Mu, a comprehensive Chinese Compendium of Materia Medica published in 1578, states ‘basket clams can prevent alcoholism and cholestasis’^6^. In Japan, Shokuhin yamato-uta, a collection of ‘waka’ poems about foods published in 1787 states ‘basket clams cure jaundice’^7^. The consumption of basket clams is recommended after the intake of excessive amounts of alcohol in Japan. Recent studies have shown that basket clams possess cholesterol-lowering^8–10^, cancer-suppressing^11,12^, and hepatoprotective properties^13^.

**Figure 1:**
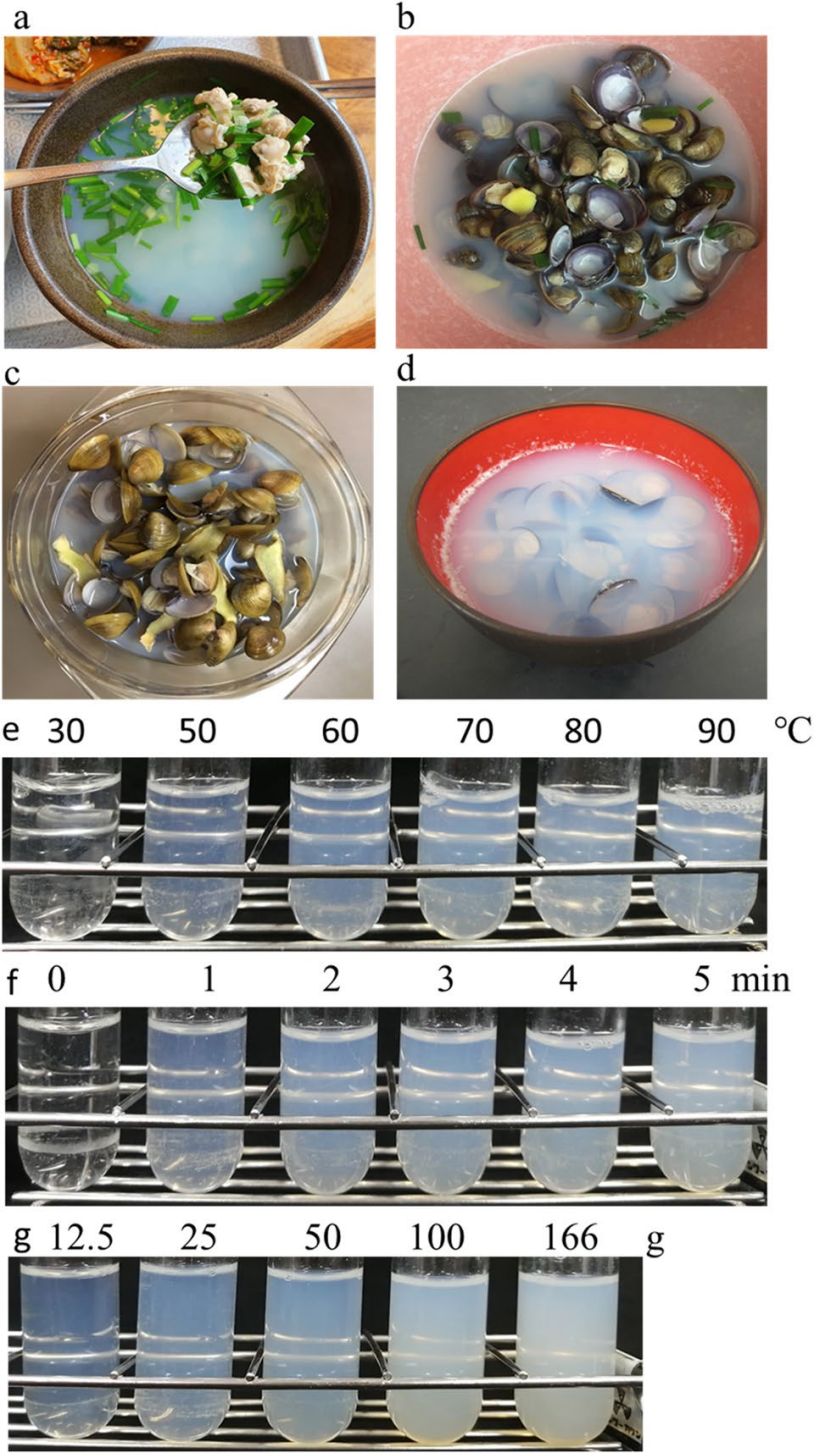
Soup with basket clam. (**a**) South Korean soup *(jaechup-guk*). (**b**) Chinese soup (*xiǎn bèi jiàng tang*). (**c**) Taiwanese soup (*xiǎn tang*). (**d**) Japanese soup (*shijimi-jiru*). (**e**) One hundred grams of sample placed in 200 mL of water was heated at 30, 50, 60, 70, 80, and 90 °C for 30 min. (**f**) One hundred grams of sample suspended in water was boiled for 0, 1, 2, 3, 4, and 5 min. (**g**) 12.5, 25, 50, 100, and 166 g of sample suspended in 100 mL of water were boiled for 3 min.

In Asian countries, the basket clams are mainly used as an ingredient in clam soups. Basket clam soup (*jaechup-guk*) is a South Korean delicacy (Fig. 1a) and is served in many specialty restaurants for basket clams in South Korea. The basket clams are also used in soups in China and Taiwan (Fig. 1b,c). Clam extract drinks (*xian jing*) are sold in Taiwan, and clear soup and miso soup (*shijimi-jiru*) prepared using Japanese basket clam (*C. japonica*) are popular in Japan (Fig. 1d).

Typically, the basket clams are boiled in hot water before consumption, and boiling causes the clam soup to turn white. It is considered that higher the cloudiness of the soup, the better it tastes. Additionally, the cloudiness increases with the number of clams used for cooking. Nevertheless, the component responsible for the white colour of the boiled soup remains unidentified. Basket clams are rich in amino acids, such as alanine, glycine, and glutamic acid, and organic acids, such as succinic acid^14,15^, which are speculated to contribute to the enhanced taste of the soup. However, high concentrations of these substances do not cloud the soup. Therefore, it is unlikely that these substances cause cloudiness. Hence, the relationship between cloudiness and good taste remains unclear.

In the present study, we aimed to investigate and identify the component responsible for the white colour of Japanese basket clam and other shellfish soups.

## Methods

### Materials used

Japanese basket clam (*C. japonica*), blood clams (*Anadara broughtonii*), Asiatic hard clam (*Meretrix lusoria*), Japanese littleneck (*Ruditapes philippinarum*), Japanese oysters (*Crassostrea nippona*), hard clam (*Mercenaria mercenaria*), Japanese scallop (*Mizuhopecten yessoensis*), Mediterranean mussel (*Mytilus galloprovincialis*), banana prawn (*Fenneropenaeus merguiensis*), and snow crab (*Chionoecetes opilio*) were obtained from a supermarket in Shimane Prefecture, Matsue City.

### Formation of cloudy liquid

To investigate the optimal extraction temperature, 100 g of the sample clams placed in 200 mL of water was heated at 30, 50, 60, 70, 80, and 90 °C for 30 min. To investigate the optimal cooking time, 100 g of the sample clams placed in 200 mL of water was boiled for 0, 1, 2, 3, 4, and 5 min. To investigate the optimal number of clams required to obtain the highest degree of cloudiness in the soup, 12.5, 25, 50, 100, and 166 g of sample clams were placed in 100 mL of boiling water for 3 min. The treated samples were filtered using a filter paper (Qualitative filter papers No. 2; Advantec, Tokyo, Japan) and centrifuged at 8000×*g* for 10 min at 4 °C.

### Ultrafiltration

The treated samples were subjected to ultrafiltration using an Amicon Pro Purification System with a 100-kDa cut-off (Merck Millipore, Tokyo, Japan) upon centrifugation at 8000×*g* for 90 min at 4 °C.

### Ninhydrin reaction

Equal volumes of 1% ninhydrin solution (Fujifilm Wako Pure Chemical, Osaka, Japan) were added to the obtained treated samples, heated in a boiling water bath for 10 min and subsequently allowed to cool to 25 °C. The colour formation was examined.

### Trichloroacetic acid (TCA) precipitation

Sample and 20% TCA were mixed in equal amounts, stirred, placed on ice for 10 min, and centrifuged at 10,000×*g* for 10 min at 4 °C.

### Tyndall effect

A laser pointer (635 nm, 200-LPP029; Sanwa Supply Inc., Okayama, Japan) was used to observe the Tyndall effect.

### Electron microscopy

The boiled soup was stained using a negative staining method. Briefly, one drop of the soup was placed on a microgrid covered with a carbon support film and hydrophilised via ion sputtering. The microgrid was allowed to absorb the sample for 10 min; then, 2% solution of uranyl acetate was added on the microgrid, and excess solution was removed using a filter paper. The microgrid was air dried and imaged using a transmission electron microscope (EM-002B; TOPCON Co., Ltd., Tokyo, Japan) at an acceleration voltage of 80 kV.

### Sodium dodecyl sulphate–polyacrylamide gel electrophoresis (SDS-PAGE) and enzymatic proteolysis

Protein concentrations were measured using the Bradford method, with bovine serum albumin (Fujifilm Wako Pure Chemical, Osaka, Japan) used as the standard. Proteins (5 µg) were separated on a 10% (w/w) polyacrylamide gel at a constant current of 25 mA/gel and stained for 60 min with Coomassie Brilliant Blue R-250 (Fujifilm Wako Pure Chemical) and acetic acid solution for 60 min. Depending on the volume of the gel pieces, 2‒5 µL of trypsin (50 ng/µL) (Promega Madison, WI, USA) was added for protein digestion at 37 °C for 16 h.

### Matrix-assisted laser desorption/ionisation-time-of-flight tandem mass spectrometry (MALDI-TOF–MS/MS)

The trypsin-digested samples were subjected to 1:5 dilution in 0.1% trifluoroacetic acid (TFA) dissolved in water. The reaction mixture was then loaded onto C18 pipette tips (ZipTip; Millipore, USA) and was passed through 10 times. The reaction mixture was subsequently washed with 0.1% TFA in water, and peptide fractions were eluted with 50% acetonitrile solution. The eluted fractions were then mixed with α-cyano-4-hydroxycinnamic acid (Fujifilm Wako Pure Chemical) in 60% (v/v) acetonitrile and 1% (v/v) TFA, and applied on a mass spectrometry (MS) target plate. MS data were obtained using a 5800 MALDI-TOF/TOF analyser (ABSciex, Concord, Canada) according to the manufacturer’s instructions. A monoisotopic precursor for MS/MS was interpreted via automatic precursor selection using DynamicExit algorithm (AB Sciex). The MS/MS data were analysed using ProteinPilot™ software (version 3.0) and the Paragon protein database search algorithm (AB Sciex). Each MS/MS spectrum was searched using the database constructed by AB Sciex.

### Absorption spectroscopy

Ultraviolet–visible absorption spectrum was obtained in the range 200–300 nm using a NanoDrop 1000 spectrophotometer (Thermo Fisher Scientific, Cleveland, OH, USA).

### Element estimation using inductively coupled plasma (ICP)-MS analysis

The boiled soup was filtered using a 100-kDa ultrafiltration spin column and precipitated using TCA; the pellet was treated with nitric acid and then analysed using ICP-MS. The concentration of S, Na, Mn, Ti, As, Fe, Ni, Cu, Li, Mg, Al, Si, P, K, Ca, Co, Zn, Rb, and Cs was determined using ICP-MS (8800 Triple Quadrupole ICP-MS; Agilent, CA, USA), following the manufacturer’s instructions.

### Fatty acid analysis using gas chromatography‒mass spectrometry (GC–MS)

Fatty acids in the soup were extracted using chloroform. All fatty acids were derivatised to volatile methyl esters using esterification kits (Nacalai Tesque Inc.), following the manufacturer’s instructions. The gas chromatography analysis of methylated fatty acids was performed on a Shimadzu QP2010 quadrupole Gas Chromatography Mass Spectrometry (GC–MS) instrument equipped with a carbowax (30 m × 0.25 mm ID; 0.25 mm film thickness) capillary column (intercut DB5MS; Agilent Technologies, CA, USA).

### Isolation of the tropomyosin-coding gene from *C. japonica*

The total RNA was extracted from *C. japonica* using the ISOSPIN Plant RNA kit (Nippon Gene, Tokyo, Japan) and digested with DNase I (NipponGene Co., Ltd., Tokyo, Japan) according to the manufacturer’s instructions. A 3-µg aliquot of total RNA was used to synthesise single-stranded cDNA using the ReverTra Ace qPCR RT master mix (Toyobo Co., Ltd., Osaka, Japan) according to the manufacturer’s instructions. For PCR amplification, the cDNA was denatured at 98 °C for 3 min in the first cycle and then for 10 s in the subsequent cycles. Primer annealing and template extension were performed at 55 °C for 5 s and 72 °C for 10 s, respectively. Thirty cycles of PCR were performed using the following oligonucleotide primers: 5′-GCCATCAAGAAGAAGATGCAGGCAATGG-3′ and 5′-CCAGCCAATTCAGCAAA-3′. The PCR products were fractionated on a 1% (w/v) agarose gel, and DNA fragments from agarose gel slices were purified using a DNA fragment purification kit (MagExtractor, Toyobo Co., Ltd.). The purified reverse transcription-polymerase chain reaction (RT-PCR) products were directly sequenced using the BigDye termination kit ver 3.1 (Applied Biosystems, Foster City, CA, USA).

### Construction of recombinant tropomyosin in *Escherichia coli*

The *C. japonica* tropomyosin gene was PCR amplified using appropriate primers (5′-CATCATCATCATCATATGGATGCCATCAAGAAGAA-3′ and 5′-TATCTAGACTGCAGGTTAATACCCAGCCAATTCAG-3′). The amplicons were cloned into the pColdII expression vector to create pColdII*-*tropomyosin with a His-tag using the In-fusion^®^ Snap Assembly master mix (Takara Bio Inc., Shiga, Japan) according to the manufacturer’s instructions. The construct was sequenced for confirmation and then transformed into *E. coli* BL21 (DE3) cells for the production of His_6_-tagged (N-terminus) *C. japonica* tropomyosin.

### Purification of the recombinant proteins

The transformants were cultured at 37 °C in Luria–Bertani medium containing ampicillin (50 µg/L) (Fujifilm Wako Pure Chemical) until the OD_600_ was approximately 0.5. Next, the temperature was decreased to 15 °C to induce cold-shock promoters, and incubation was continued for 24 h. The cells were harvested via centrifugation (6000×*g*, 10 min, 4 °C) and suspended in 1 mL of protein extraction reagent (Integral Co., Tokushima, Japan). One microliter of 1000 U/mL recombinant DNase I (Takara Bio Inc. Shiga, Japan) and 0.2 mg/mL lysozyme (Fujifilm Wako Pure Chemical) were added to each sample and incubated for 15 min at room temperature. Thereafter, the samples were centrifuged (13,000×*g*, 20 min, 4 °C) and recombinant proteins were isolated using the His-Spin protein miniprep kit (Zymo Research Co., Irvine, CA, USA), following the manufacturer’s instructions.

### Elemental analysis of tropomyosin using ICP-MS

The boiled soup was purified using an Amicon Pro Purification System with a 100-kDa cut-off (Merck Millipore. MA, USA). The samples were collected following TCA precipitation. Subsequently, nitric acid was added to the pellet, and the mixture was incubated at 100 °C for 12 h. The degraded product was suspended in 0.1 N nitric acid solution and analysed using ICP-MS (8800 Triple Quadrupole ICP-MS; Agilent, CA, USA).

### Amino acid composition of all human proteins

Amino acid sequence data of all human proteins were downloaded from Uniprot (https://ftp.uniprot.org/pub/databases/uniprot/current_release/knowledgebase/reference_proteomes/Eukaryota/UP000005640/UP000005640_9606.fasta.gz). Only the canonical entries were used for the following analysis. The amino acid composition was calculated using the Perl script (https://www.nntp.perl.org/group/perl.beginners/2010/12/msg114941.html) with a slight modification (Supplementary Data 1).

## Results

### Identification of white ingredients in boiled Japanese basket clam soup

**Figure 2:**
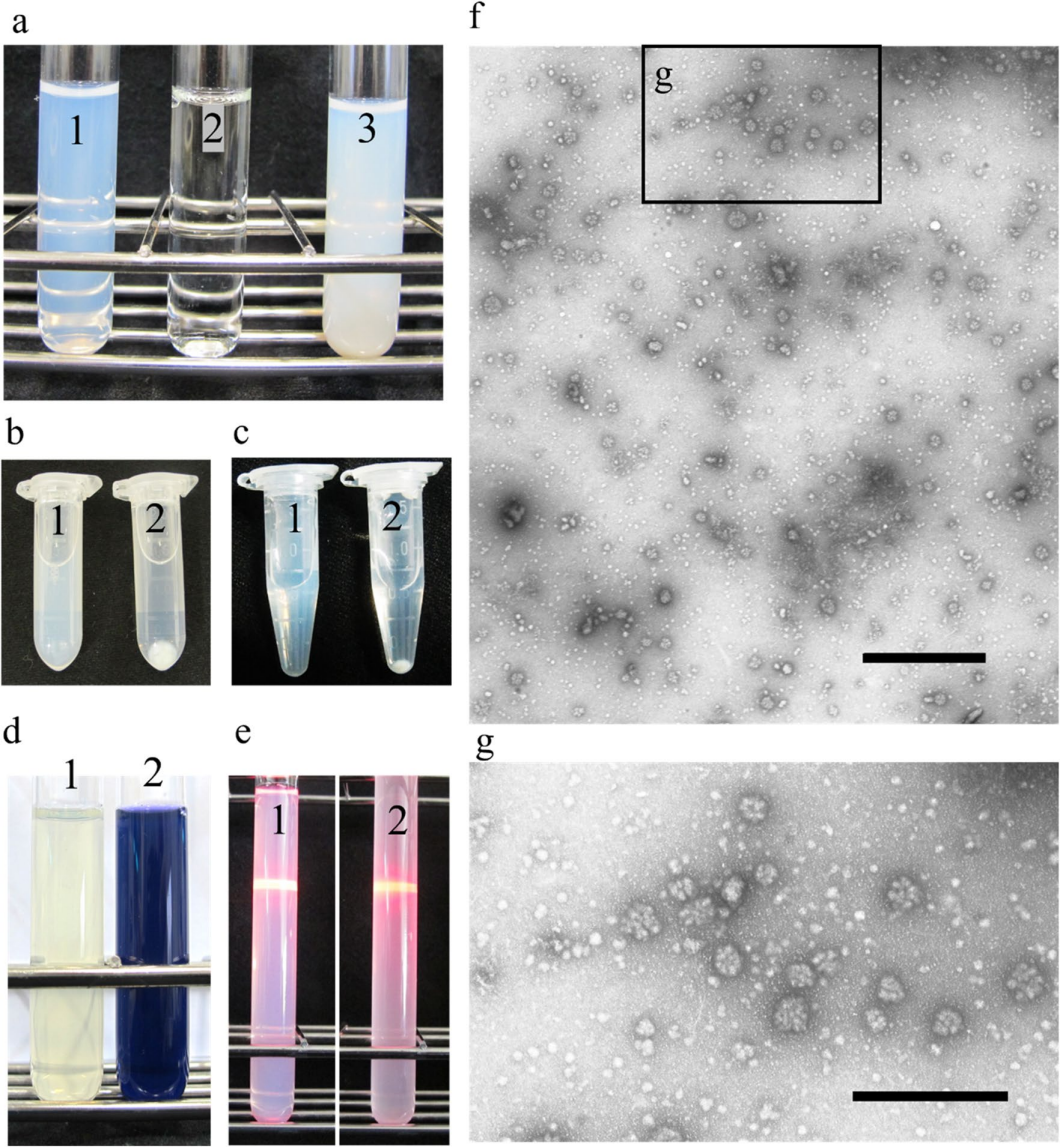
Analysis of components that whiten boiled clam soup. (**a**) Boiled soup of basket clam (1); ultrafiltered flow fraction (2); ultrafiltered supernatant (3); (**b**) TCA was added to a final concentration of 10% to boiled basket clam soup (1) and centrifuged (2). (**c**) Ammonium sulphate was added to a final concentration of 60% to boiled basket clam soup (1) and centrifuged (2). (**d**) Ninhydrin was added to a final concentration of 1% to boiled basket clam soup (1) and heated (2). (**e**) Milk was diluted 1:10 with distilled water and irradiated with a laser beam (1); irradiated boiled basket clam soup (2). (**f**) Negative stain image of boiled soup in transmission electron microscopy. Bar: 1 µm. (**g**) Enlarged image of particles. Bar: 0.5 µm.

To determine the optimal extraction temperature, the samples were heated at 30, 50, 60, 70, 80, and 90 °C for 30 min. The water started to turn cloudy at around 50 °C, and at around 70 °C, the cloudiness peaked (Fig. 1e). To determine the optimal cooking time, the clam samples were boiled at different time points. The cloudiness peaked after 2 min of cooking (Fig. 1f). To determine the optimal number of clams required to obtain the highest degree of soup cloudiness, different numbers of clam samples were placed in boiling water for 3 min. The degree of cloudiness increased with the number of clams (Fig. 1g). To investigate the size of the component responsible for soup cloudiness, ultrafiltration was performed using a 100-kDa ultrafiltration spin column (Fig. 2a). The obtained flow-through fraction was transparent (Fig. 2b) and was eluted as a large macromolecule (Fig. 2c), suggesting the presence of a protein. Therefore, we precipitated it using TCA or ammonium sulphate, followed by centrifugation. The obtained supernatant was transparent (Fig. 2b,c), but the precipitated pellet turned white when dissolved in water (data not shown). The boiled soup turned blue when heated with ninhydrin, a reagent used for amino acid detection (Fig. 2d). Streaks of light were visible when the solution was irradiated with a laser (Fig. 2e). Based on the results obtained, we speculated that the protein formed colloids in this white liquid. Lipids are known to form emulsions that make the aqueous solution cloudy. To investigate the involvement of lipids in soup cloudiness, fatty acid concentration was measured using GC–MS and estimated to be 5.7 µg/mL (Supplementary Table 1a). Compared to the concentration of proteins obtained in the soup (989 µg/mL), the concentration of fatty acids detected was not considered to play a major role in soup cloudiness.

Electron microscopy observations of the negatively stained boiled soup indicated the presence of numerous colloidal particles. The diameter of the smallest colloidal particles was 16.0 ± 3.2 nm (n = 44), whereas that of the largest particles was 91.7 ± 19.5 nm (n = 45) (Fig. 2f,g). These particles appeared smaller than the micelles found in milk^16^, of size approximately 170 nm and diameter 206 nm, and predominantly made of casein.

**Figure 3:**
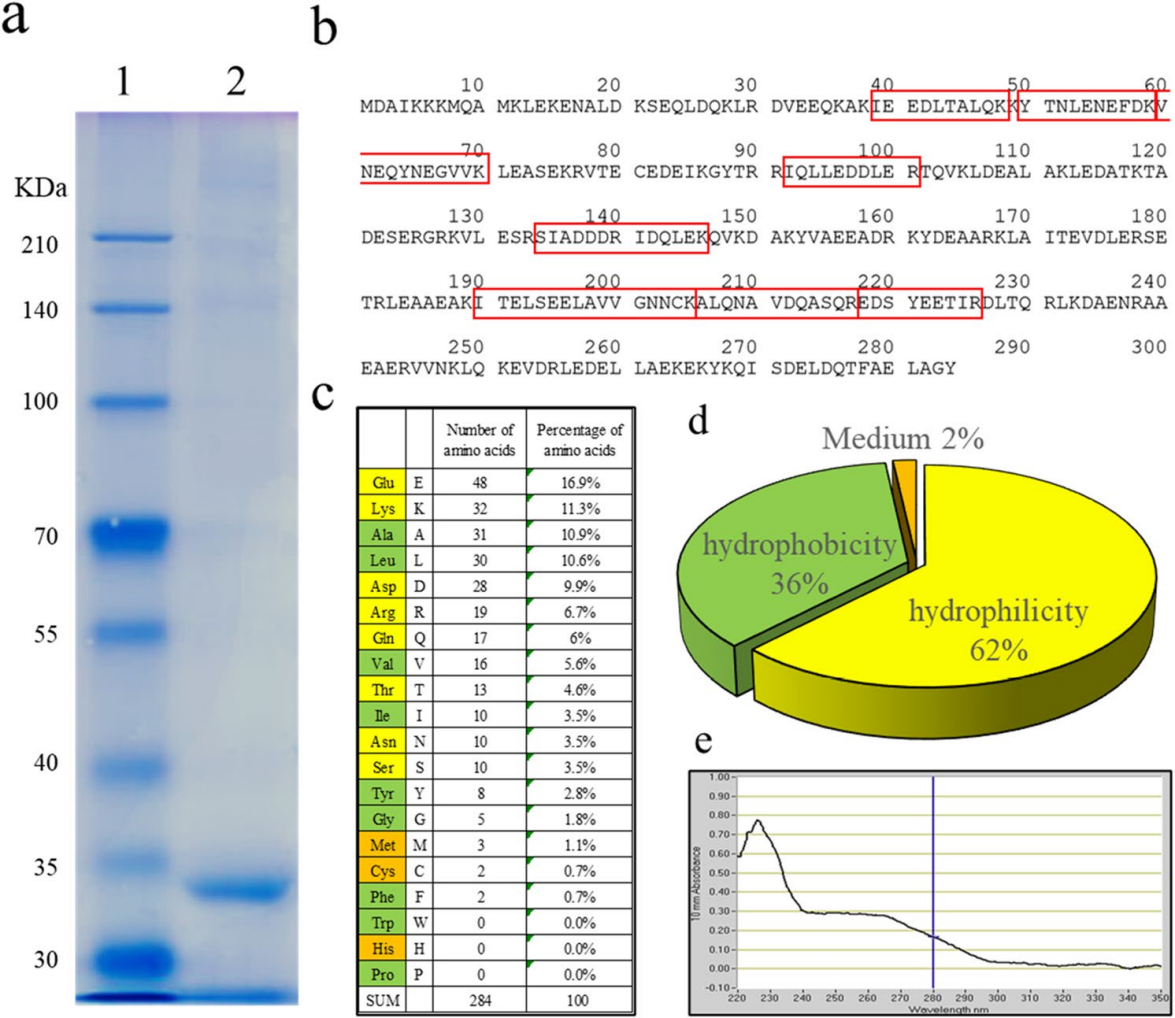
Identification of proteins using SDS-PAGE and MALDI-TOFMSMS, and amino acid composition of the characterised proteins. (**a**) SDS-PAGE of boiled basket clam soup. (**b**) Deduced amino acid sequence of Japanese basket clam (*Corbicula japonica*) tropomyosin. Amino acids surrounded by red boxes are the parts detected using MALDI-TOF–MS/MS. (**c**) Amino acid composition of Japanese basket clam (*C. japonica*) tropomyosin. (**d**) Ratio of hydrophilic/hydrophobic/amphoteric amino acids. (**e**) UV absorption of basket clam extract from 220 to 350 nm.

To investigate the type of protein present in the boiled soup, the protein was subjected to SDS-PAGE. From the results of ultrafiltration experiments, the size of the intended material was estimated to be larger than 100 kDa. SDS-PAGE did not reveal any products larger than 100 kDa, but a prominent band appeared at approximately 33 kDa (Fig. 3a). The 33-kDa band was recovered from the gel, digested with trypsin, and then subjected to peptide mass fingerprinting using MALDI-TOF–MS/MS. The results revealed the molecule as tropomyosin (accession number P43689) of *Biomphalaria glabrata*, a freshwater snail (Supplementary Fig. 1a). With no data on the amino acid sequence of Japanese basket clam (*C. japonica*) tropomyosin, the cDNA of tropomyosin was PCR amplified and sequenced, as shown in Fig. 3b. Japanese basket clam tropomyosin consists of 284 amino acid residues, with an estimated molecular weight of 32.8 kDa (accession number LC564862). Theoretical pI/MW (average) of Japanese basket clam tropomyosin is 4.63/32,812.40 Da. Interestingly, tropomyosin extracted from Japanese basket clam contains only 17 types of amino acids, wherein tryptophan, histidine, and proline residues are absent and only two residues of cysteine and phenylalanine are present (Fig. 3c). The tropomyosin sequence revealed the presence of 48 glutamic acid (16.9%), 32 lysine (11.3%), 30 leucine (10.5%), 31 alanine (10.9%), and 28 aspartic acid (9.8%) residues (Fig. 3c). The proportion of hydrophilic amino acids in tropomyosin was high at 62% (Fig. 3d). Amino acids with aromatic side chains, such as tryptophan, tyrosine, and phenylalanine, exhibit absorption spectra at 230–300 nm. Due to the absence of tryptophan, with the highest absorbance at 230‒300 nm, the absorbance of Japanese basket clam tropomyosin was measured at 200–360 nm instead. No peak was detected at 280 nm, the maximum wavelength of absorption by the protein, but a peak was detected at 200–215 nm, the maximum absorption wavelength of peptide bonds (Fig. 3e).

The MALDI-TOF–MS/MS data were reanalysed using the determined amino acid sequence of Japanese basket clam tropomyosin, and data obtained were consistent with the estimated presence of eight peptides (Fig. 3b, coloured red square, Supplementary Fig. 1b,c). Hence, based on the results obtained, we concluded the detected protein to be tropomyosin.

**Figure 4:**
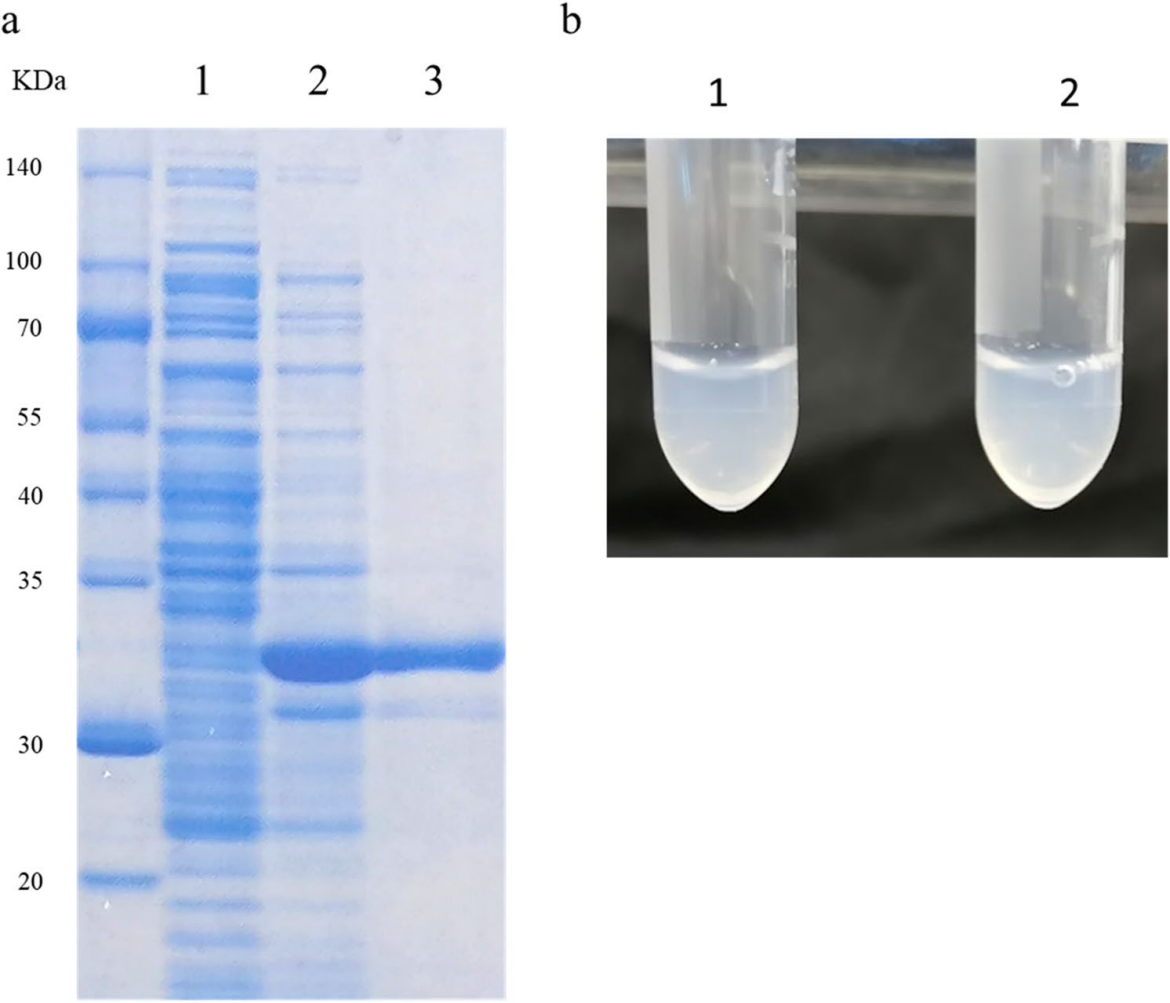
Expression and purification of *Escherichia coli* tropomyosin. (**a**) Tropomyosin from Japanese basket clam (*Corbicula japonica*) was expressed in *Escherichia coli*, and the protein was extracted, purified, and subjected to SDS-PAGE. (1) Protein extracted from *Escherichia coli* under non-induced conditions. (2) Protein extracted from *E. coli* under induced conditions. (3) Purified His-tag protein. (**b**) (1) His-tag purified protein extracted from *E. coli.* (2) His-tag purified protein extracted from *E. coli* boiled for 5 min.

To further validate the involvement of tropomyosin in soup cloudiness, we expressed it in *E. coli* using a protein expression vector (pCOLD2). The expressed protein was purified (Fig. 4a) and suspended in water that turned the solution cloudy (Fig. 4b). The cloudiness of the solution persisted even upon boiling at 100 °C for 3 min (Fig. 4b). Overall, we confirmed the involvement of tryptophan in the cloudiness of the boiled soup.

### Characteristics of Japanese basket clam tropomyosin

Calcium ions and phosphate are involved in the formation of casein micelles in milk^17^. To investigate whether calcium and phosphate are involved in the formation of micelles in Japanese basket clam soup, acid-degraded samples was analysed using ICP‒MS. We did not detect calcium and phosphate ions. As Japanese basket clam tropomyosin contains three residues of methionine, a sulphur-containing amino acid, the amount of protein can be estimated by measuring the amount of sulphur. As expected, sulphur and chloride were detected using ICP‒MS; however, chloride was detected at lower levels and was only about one-thirteenth the level of sulphur (Supplementary Table 1b). This finding suggests no involvement of these elements in the formation of micelles.

To determine whether the prevention of hydrophobic bond formation in tropomyosin micelles affects cloudiness, SDS was added to the boiled soup of Japanese basket clam and heated, and there was no change in the cloudiness (Supplementary Fig. 2a). The skim milk powder, when dissolved in water, appears cloudy (Supplementary Fig. 2b). We compared the disappearance of cloudiness between clam soup and skim milk solution by adding SDS to skim milk solution. Interestingly, the cloudiness of the skim milk solution decreased upon the addition of SDS, and the solution became almost transparent upon boiling (Supplementary Fig. 2b); whereas, the extent of clam soup cloudiness did not change, even upon the addition of β-mercaptoethanol to disrupt the S‒S bond (Supplementary Fig. 2c), or urea or guanidine hydrochloride to denature proteins (Supplementary Fig. 2c3,c4). SDS-PAGE performed using boiled soup treated with SDS and β-mercaptoethanol did not show the disappearance of cloudiness, but still showed the band of size approximately 33 kDa. Upon the addition of organic solvents, such as methanol and acetone, to the boiled soup, a white layer was observed (Supplementary Fig. 2d), which turned cloudy when chloroform was added (Supplementary Fig. 2d). Next, we investigated whether the extent of cloudiness varies with pH, and we observed that precipitation occurred in the pH range of 3–5. The solution became transparent at pH below 3, and the cloudiness almost disappeared at pH 1 (Supplementary Fig. 2e). These results indicate that the micelles of tropomyosin are heat resistant and are not denatured upon the addition of denaturants such as SDS, β-mercaptoethanol, urea, and guanidine hydrochloride, but can be denatured by organic solvents such as methanol, acetone, chloroform, and strong acids.

**Figure 5:**
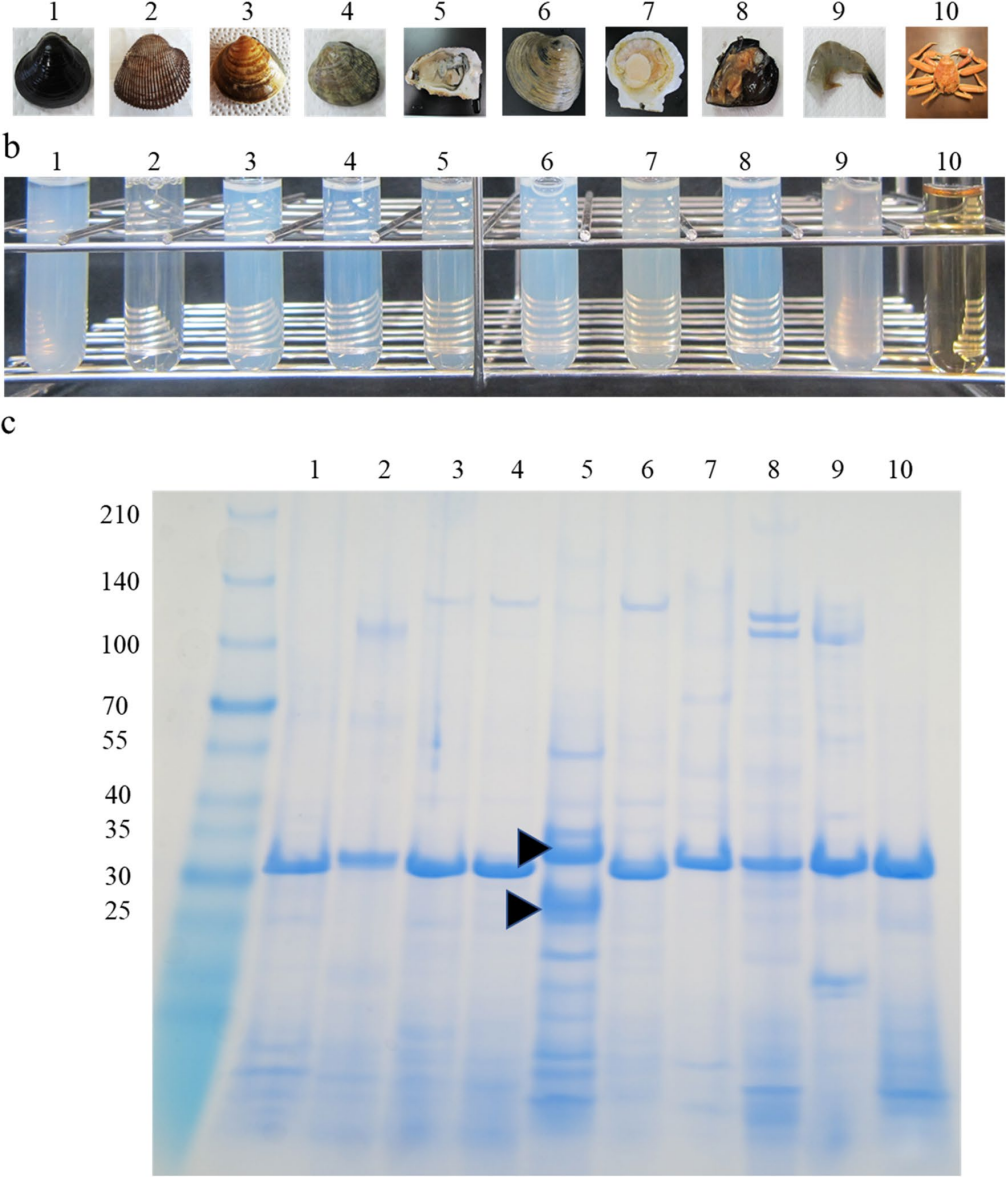
Boiled soups of various shellfish, shrimp, and crab, and SDS-PAGE analysis of the boiled soups. (**a**) Photograph of samples. (**b**) Boiled soups. (**c**) SDS-PAGE of boiled soups. (1) Japanese basket clam (*Corbicula japonica*). (2) Bloody clam (*Anadara broughtonii*). (3) Asiatic hard clam (*Meretrix lusoria*). (4) Japanese littleneck (*Venerupis philippinarum*). (5) Pacific oyster (*Crassostrea gigas*). (6) Hard clam (*Mercenaria mercenaria*). (7) Japanese scallop (*Patinopecten yessoensis*). (8) Mediterranean mussel (*Mytilus galloprovincialis*). (9) White leg shrimp (*Litopenaeus vannamei*). (10) Snow crab (*Chionoecetes opilio*). The arrows indicate the bands used in MALDI-TOFMS/MS analysis, namely the upper and lower bands.

Similar results were obtained in shellfish, such as blood clam, Asiatic hard clam, Japanese littleneck clam, Japanese oyster, hard clam, Japanese scallop, and Mediterranean mussel (Fig. 5a,b). As tropomyosin in crustaceans (shrimp and crab) has been shown to be heat resistant^14^, a similar set of experiments was performed on banana shrimp and snow crab. As expected, no cloudiness was observed in the snow crab soup (Fig. 5b). Furthermore, cloudiness was not observed when similar experiments were performed with chicken (*Gallus domesticus*) and fish (*Doederleinia berycoides*) soups (data not shown). SDS-PAGE of protein extracted from the boiled soup revealed an intense band of approximate size 33–37 kDa for all examined organisms, except for Japanese oyster soup that showed two intense bands instead (Fig. 5c; Supplementary Fig. 1d,e). Similar results have been reported for raw oyster (*Crassostrea belcheri*)^15^, wherein the 37-kDa protein was identified as tropomyosin, but the identification of the other band needs further investigation^15^. Notably, the MALDI‒TOF‒MS/MS and Protein Pilot™ (AB Sciex) analyses revealed that the proteins investigated from all examined organisms in this study to be tropomyosin.

## Discussion

The basket clam is a popular food in Asia. Boiled basket clam soup is white and cloudy, and the components contributing to its colour are still unknown. In this study, we showed that the whitening of the boiled soup is due to the presence of tropomyosin forming micelles. Japanese basket clam tropomyosin expressed in *E. coli* made water cloudy, and a low proportion (1/200 of the proteins) of fatty acids was found compared to that of proteins. These results indicate that tropomyosin is the major component contributing to the cloudiness of the clam soup. Furthermore, similar results obtained for other shellfish further validated the involvement of tropomyosin in the cloudiness of boiled shellfish soup. To investigate the involvement of tropomyosin in taste enhancement, the soup was tasted after the removal of amino acids and citric acid (the known taste enhancers) using ultrafiltration. The resulting product was bitter and lacked umami flavour (results not shown), suggesting that tropomyosin does not enhance the taste of the soup.

Proteins are usually denatured, precipitated, or degraded upon boiling; however, the basket clam tropomyosin’s ability to form micelles in water prevents its denaturation on boiling. Therefore, the cloudiness of the soup is not affected even upon boiling for 24 h. Furthermore, these tropomyosin micelles did not precipitate when stored at a low temperature of 4 °C for up to 6 months. Tropomyosin from crabs and shrimp has been reported to be thermostable^18^, and that from birds (chicken) shows a three-dimensional coiled-coil structure, with the α-helix content close to 100%^19^. Proteins that are rich in α-helices have been investigated using statistical tools^20^. Amino acids with high α-helix-forming ability, including glutamate, methionine, alanine, and leucine, are expected to stabilise the α-helix^21^ and, thus, the entire protein structure. The proportion of these amino acids in tropomyosin was estimated to be 39.5%, whereas that of hydrophilic amino acids was 62% (Fig. 3d). Unlike that of glycine, the *R*-group of tryptophan and tyrosine is bulky and destabilises the α-helices. Proline also destabilises the α-helix owing to its irregular shape^22^. The *R*-group of proline binds to the nitrogen of the amide group, which causes steric hindrance. Hydrophobic tryptophan, proline, and neutral histidine were not detected among the 284 amino acid residues of Japanese basket clam tropomyosin. When we examined 14,508 human proteins containing more than 280 amino acid residues, only 12 proteins, including keratin-associated protein and basic salivary proline-rich protein, consisted of 17 or fewer amino acids (constituent amino acids) (Supplementary Table 2). All these proteins contained tryptophan, proline, or histidine. These results show that tropomyosin from Japanese basket clam, which is rich in hydrophilic amino acids and contains many amino acids with high α-helix-forming ability, has an extremely rare amino acid composition that contributes to its solubility in water.

This study revealed that tropomyosin from Japanese basket clam is resistant to denaturants. The turbidity of the clam soup persisted even when boiled with SDS and β-mercaptoethanol, and SDS-PAGE revealed only one protein band of size 33 kDa. The mechanism by which the micelles undergo decomposition/denaturation needs further investigation. Although boiled snow crab soup did not show cloudiness, but protein bands were detected in SDS-PAGE (Fig. 5b,c), suggesting differences in the formation and properties of tropomyosin micelles among crabs, shellfish, and shrimps. Japanese basket clam tropomyosin is denaturant resistant, thermostable, and highly hydrophilic, and proteins with such properties are used in various industries. In the future, three-dimensional structure analysis of tropomyosin micelles and amino acid substitution experiments can improve our understanding regarding this protein, which can be utilised in industries.

Tropomyosin from crustaceans, such as shrimps and crabs, has been reported to be the major allergen in shellfish^17,23^. The amino acid sequence of tropomyosin from Japanese basket clam showed 56.7% homology with that from banana shrimp and snow crab. Unlike the eight epitopes present in shrimp and crab tropomyosin^24^, only one epitope was identified in Japanese basket clam tropomyosin (Supplementary Fig. 3a, red box). The oyster (*Crassostrea gigas*) tropomyosin has been reported to be an allergen^25^. The homology between oyster tropomyosin and Japanese basket clam tropomyosin was 71%, wherein two epitopes was identified in oyster tropomyosin. Some people are allergic to Japanese basket clam^23,24^. However, no common serological and clinical cross-reactivity characteristics were observed between freshwater clams and shrimps^23^. As these results were obtained from a very small sample of only two subjects who were allergic to Japanese basket clam, further investigation is required with larger samples to examine whether tropomyosin is the major allergen in patients with Japanese basket clam allergy. In this study, just one linear epitope was identified in Japanese basket clam tropomyosin, but as conformational epitopes also exist, and they should be investigated.

Western blotting using a polyclonal antibody against full-length human tropomyosin (GenTex GTX16386) revealed a band in the protein extracted from the myocardium of mouse; however, no such band was observed in the Japanese basket clam sample (Supplementary Fig. 3b). This is possibly due to 76.4% homology observed in the amino acid sequences between human and mouse tropomyosin, compared to only 50.7% between human and Japanese basket clam tropomyosin. Tropomyosin has a periodic heptad repeat sequence characteristic of coiled-coil proteins^26^. Although the homology between human and Japanese basket clam tropomyosin is not high, the amino acids in the major sites (such as acidic residues, positively charged residues, apolar residues, D-position aspartate-137, and alanine residues) were observed to be highly conserved (Supplementary Fig. 3d). The a and d regions contained more than 70% hydrophobic amino acids, whereas the b, c, e, and f regions contained 7–32% hydrophobic amino acid residues (Supplementary Fig. 3d). The cloudiness of boiled soup of Japanese basket clam was the strongest among the soups of the examined organisms. Although the boiled soup of snow crab contained tropomyosin, the boiled soup was yellowish instead of the usual white colour of soups prepared with other examined organisms (Fig. 5b). The degree of cloudiness varied considerably from species to species (Fig. 5). When soups other than Japanese basket clam soup were observed under an electron microscope, the structure of micelles appeared to be different among the soups (data not shown). The difference in the amino acid sequence of tropomyosin may be related to the cloudiness. By conducting amino acid substitution experiments, it may be possible to identify amino acids that are important for cloudiness. Furthermore, by conducting structure analysis using higher-resolution electron microscopy, it will be possible to clarify whether the structure of micelles differs among species. Clarifying the thermostability and the mechanism underlying micelle formation would promote the application of micelles in the development of industrially beneficial thermostable proteins.

The boiled clam soup remained white and cloudy with the addition of SDS and β-mercaptoethanol even at 100 °C (Supplementary Fig. 2a). It has been established that under such conditions, tropomyosin forms micelles of size larger than 100 kDa (Fig. 2f,g). Interestingly, when separated using SDS-PAGE, a single band was observed corresponding to the 33-kDa molecular weight. However, it is not clear how electrophoresis could separate the highly stable micelles. Here, we identified for the first time, tropomyosin as the major contributing factor of cloudiness in the clam boiled soup. However, the exact reason for the marked thermostability of tropomyosin under boiling conditions remains elusive. Further research is warranted to accurately elucidate the mechanism via which tropomyosin micelles exhibit high thermostability. This knowledge can provide useful information for the production of thermostable proteins.

## Supplementary Information

Supplementary Information.

## Data Availability

The sequences of tropomyosin from various species are available at https://www.ncbi.nlm.nih.gov/. Data of all human protein sequences can be downloaded from https://ftp.uniprot.org/pub/databases/uniprot/current_release/knowledgebase/reference_proteomes/Eukaryota/UP000005640/UP000005640_9606.fasta.gz. Source data are provided with this paper.
