# Supplementary material for: Tropomyosin micelles are the major components contributing to the white colour of boiled shellfish soups
Source: Sci Rep. 2022 Sep 9;12:15253. doi: 10.1038/s41598-022-17911-8 (PMC9463138; doi:10.1038/s41598-022-17911-8)

**Supplementary figures**

**Supplementary Fig. 1**.

**a,** SDS-PAGE of Japanese basket clam boiled soup. Coomassie Brilliant Blue staining, gel purification, trypsin treatment, and analysis using MALDI-TOF-MS. The protein was identified as tropomyosin (P43689) in *Biomphalaria glabrata*.　**b, c,** Re-analysis of MALDI-TOF-MS/MS data using the amino acid sequence of tropomyosin from Japanese basket clams (*Corbicula japonica*). **b** and **c** are the results obtained using proteins purified from different bands. SDS-PAGE of **d,** Japanese oyster (upper band) and **e,** Japanese oyster (lower band) boiled soup. Coomassie Brilliant Blue staining, gel purification, trypsin treatment, and analysis using MALDI-TOF-MS. The proteins were identified as tropomyosin in *Chlamys nipponensis* and *Perna viridis,* respectively.

**Supplementary Fig. 2.**

**a**, Change in cloudiness after the addition of SDS to Japanese basket clam boiled soup. 1. White boiled soup 2. SDS (1% final concentration) was added to the boiled soup. 3. SDS (1% final concentration) was added to the boiled soup and boiled for 5 min. **b,** Change in cloudiness after the addition of SDS to 10% skim milk solution. 1. 10% skim milk solution SDS. 2. SDS (1% final concentration) was added to the skim milk solution. 3. SDS (1% final concentration) was added to the skim milk solution boiled for 5 min. **c,** Change in cloudiness when a denaturant was added to boiled soup. 1. Boiled soup, 2. β-Mercaptoethanol was added at a final concentration of 1% to the boiled soup. 3. Boiled soup treated with 3 M urea. 4. Boiled soup treated with 100 mM guanidine hydrochloride. **d,** Change in cloudiness when a denaturant was added to the boiled soup. 1. Boiled soup. 2. Acetone was added to the boiled soup. 3. Methanol was added at a final concentration of 50% to the boiled soup. 4. Chloroform was added at a final concentration of 50% to the boiled soup. **e,** Change in cloudiness of boiled soup due to variations in pH.

**Supplementary Fig, 3**.

**a,** Amino acid sequence alignments of brown shrimp and Japanese water calm tropomyosin. The epitope region is shown in a blue and red square, respectively. The part surrounded by the red square represents residues that are the same as those of Brown shrimp. **b,** Results of western blotting using Japanese basket clam boiled soup and protein extracted from mouse myocardium. SDS-PAGE and Coomassie Brilliant Blue staining. 1. Size marker; 2. Japanese basket clam soup; 3. Proteins extracted from mouse myocardium. Western blot. 4. Japanese basket clam boiled soup; 5. Mouse myocardium.  **c,** Coiled-coil helical wheel and heptad repeats. **d,** Sequence of tropomyosin from *Homo sapiens*, Japanese water clams, and white leg shrimp, emphasising the outer surface (b, c, and f) residues from the α zones (shaded). Such acidic residues (red) are found in the C-terminal half of each of the seven β zones; a positively charged residue (blue) followed by apolar residues (green) are found in the N-terminal half of most tropomyosin α-zones. The core alanine residues are highlighted in yellow, and the D-position aspartate-137 is highlighted in purple. This figure was adapted and modified from Brown *et al.*^22^ and Lehman *et al.*^23^.

1. Brown, J. H., Zhou, Z., Reshetnikova, L., Robinson, H., Yammani, R. D., Tobacman, L. S. & Cohen, C. Structure of the mid-region of tropomyosin: Bending and binding sites for actin. *Proc. Natl. Acad. Sci. USA* **102,** 18878−18883 (2005). DOI: 10.1073/pnas.0509269102.
2. Lehman, W., Rynkiewicz, M. J. & Moore, J. R. A new twist on tropomyosin binding to actin filaments: perspectives on thin filament function, assembly and biomechanics. *J. Muscle Res. Cell Motil.* **41,** 23−38 (2020). DOI: 10.1007/s10974-019-09501-5.

**Supplementary table 1.**

Elemental analysis. The Japanese basket clam boiled soup was ultrafiltered using a membrane with a 100-kDa cut-off. The supernatant was collected, TCA (final concentration, 10%) was added, the mixture was centrifuged, and the supernatant was discarded. The pellets were rinsed with water and treated with nitric acid. They were decomposed by heating at 100 °C for 12 hours. Ten millilitres of 0.1 N HNO3 was added and well mixed. These samples were used for ICP-MS analysis.

**Supplementary table 2.**

List of human proteins with less than 17 constituent amino acids. In total, 14,508 human proteins with 280 or more amino acid residues were analysed.

**Supplementary Data 1.**

Amino acid composition of all human proteins. These data were created using Perl script (https://www.nntp.perl.org/group/perl.beginners/2010/12/msg114941.html).

**Supplementary Fig. 1**

**
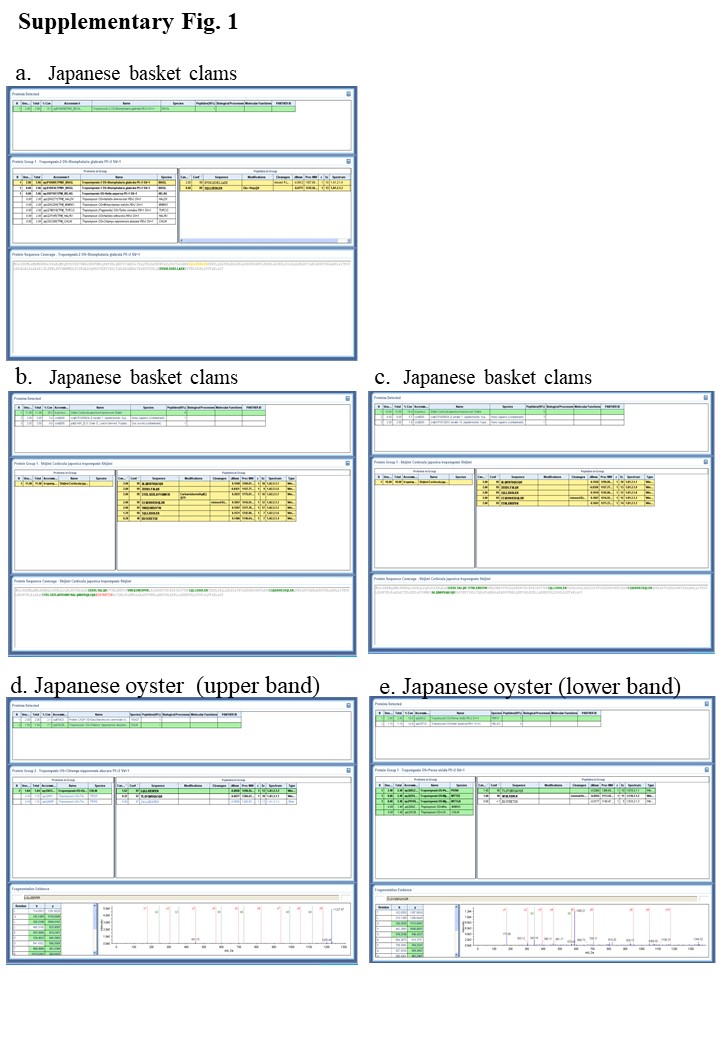
**

**Supplementary Fig. 2**.


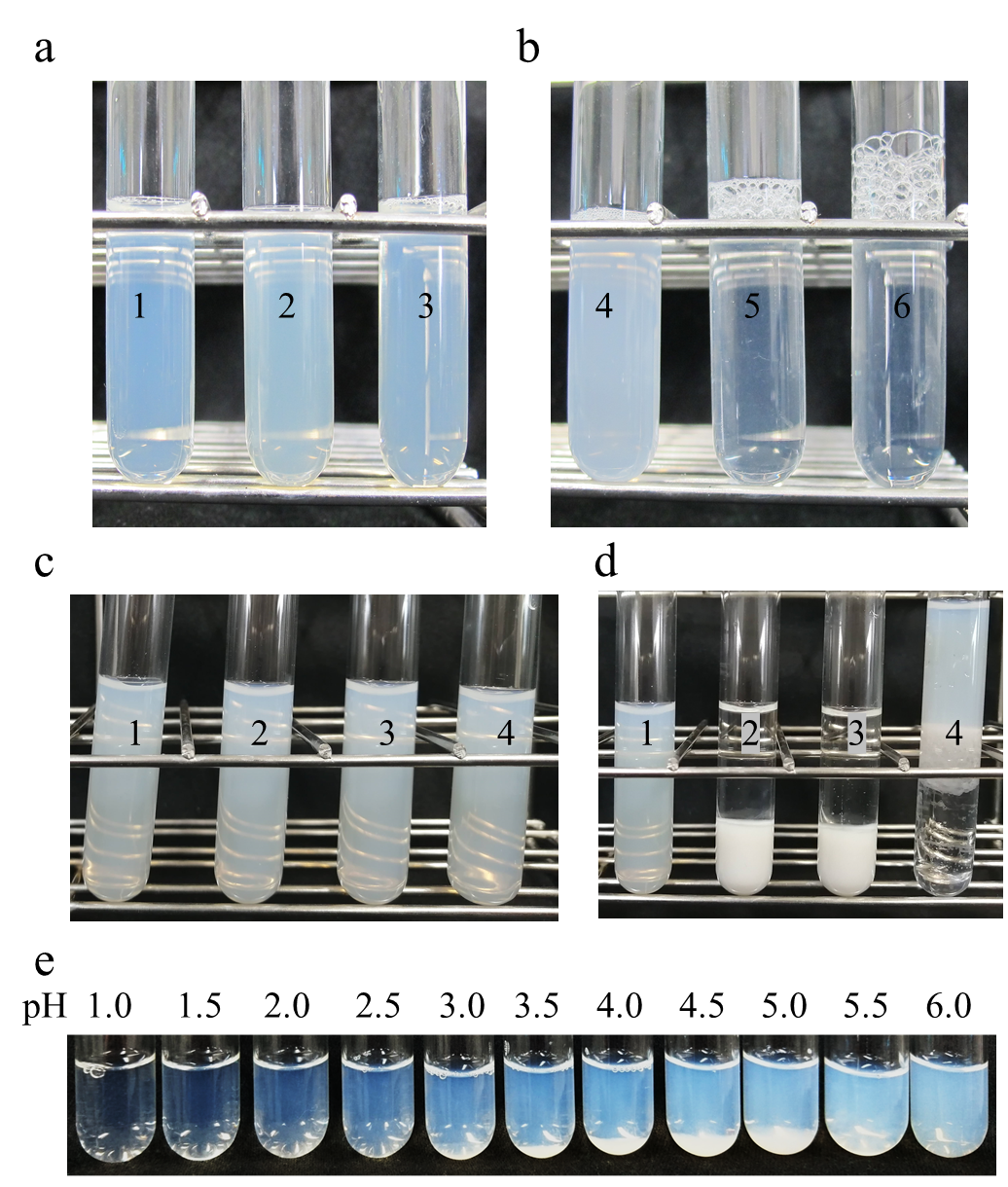


**Supplementary Fig. 3**.


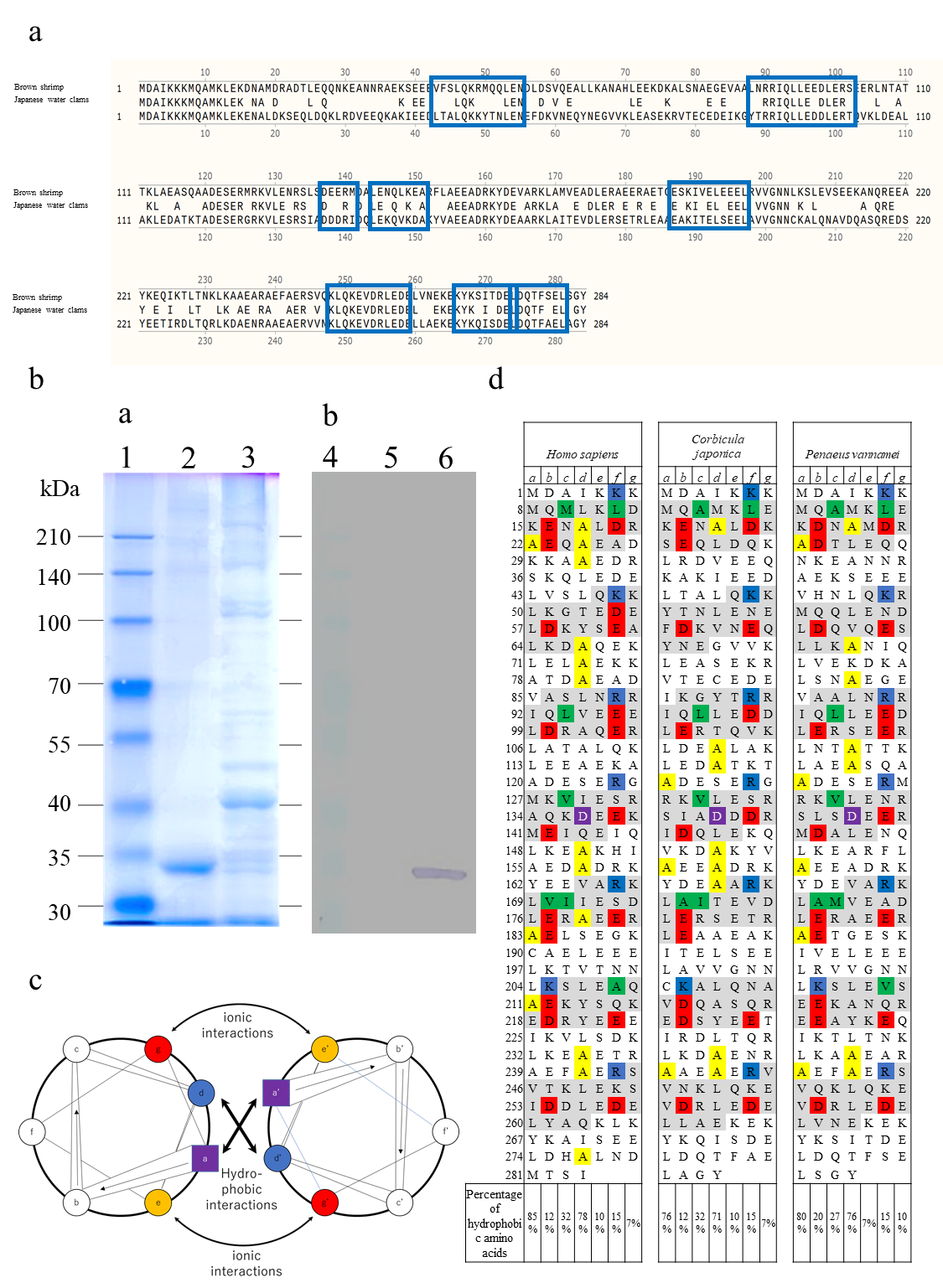


**Supplementary Table 1**


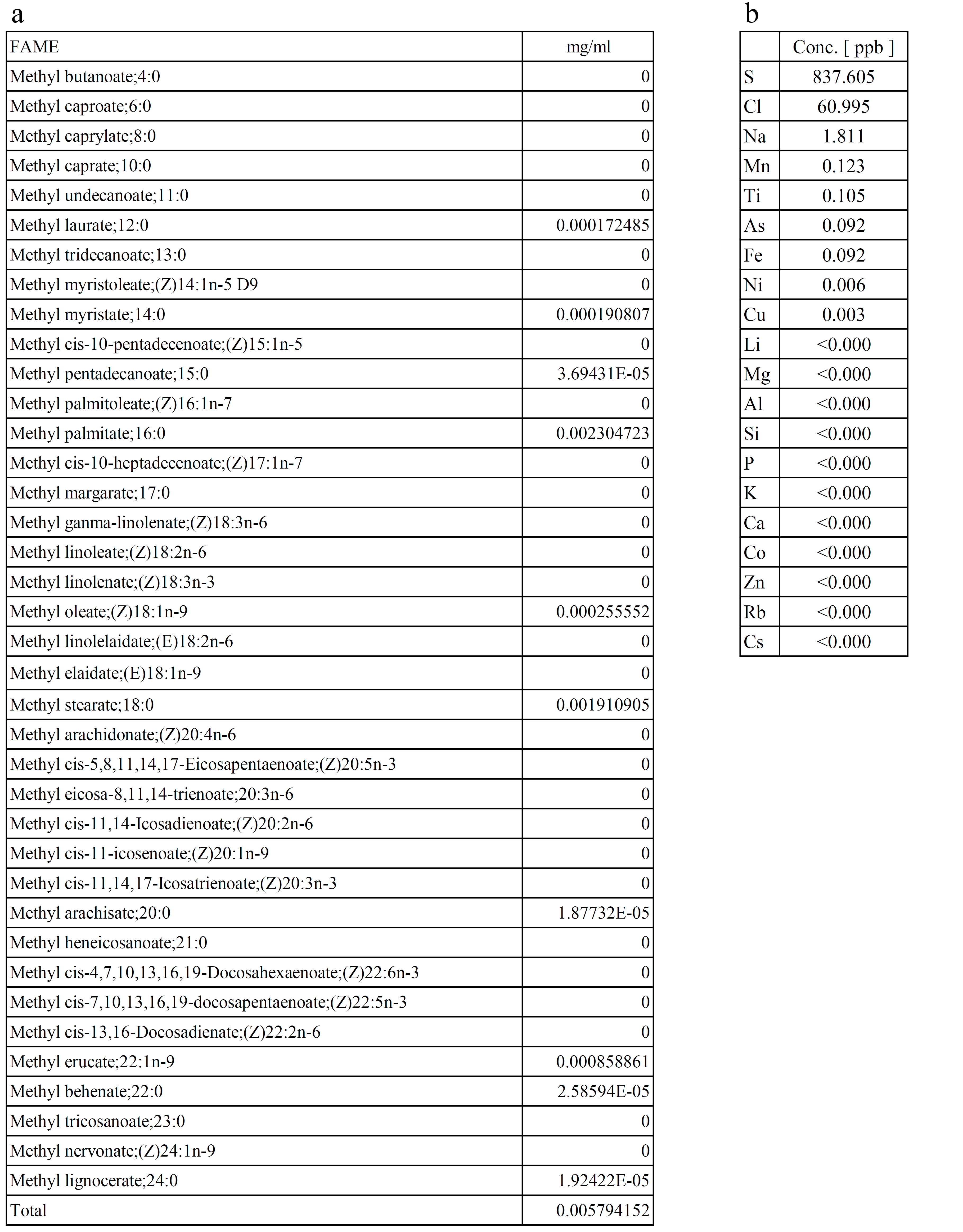


**Supplementary Table 2.**


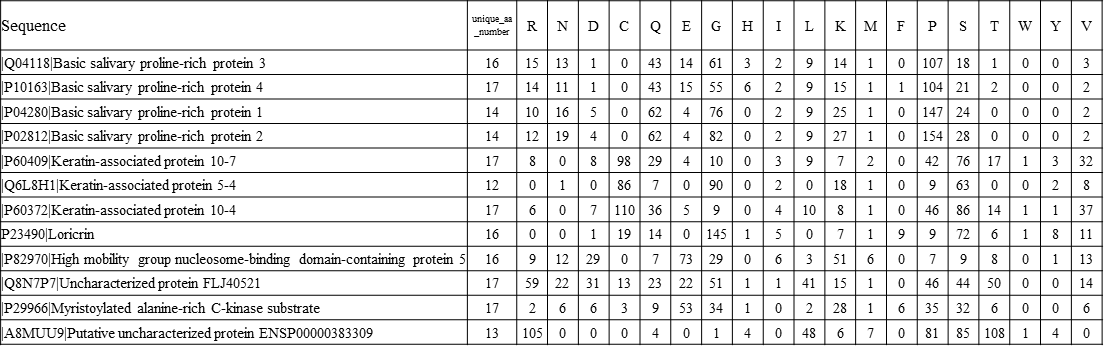

Supplement: Supplementary file 1 — Supplementary Information. [file 41598_2022_17911_MOESM1_ESM.docx]
